# An exploratory study of BLCA-4 expression, STR analysis, and urothelial cytology in smokers versus non-smokers with bladder cancer

**DOI:** 10.3389/fonc.2026.1851761

**Published:** 2026-06-17

**Authors:** Maha Abdullah Momenah, Wedad Saeed Al-Qahtani

**Affiliations:** 1 Department of Biology, College of Science, Princess Nourah bint Abdulrahman University, Riyadh, Saudi Arabia; 2 Department of Forensic Sciences, College of Criminal Justice, Naif Arab University for Security Sciences;, Riyadh, Saudi Arabia

**Keywords:** bladder cancer (BC), BLCA-4 nuclear matrix protein, short tandem repeat (STRs), smokers, urothelial cytology

## Abstract

**Background:**

Numerous studies have established that smoking is associated with a threefold increase in the risk of developing bladder cancer. However, the precise relationship between smoking and the underlying genetic and morphological alterations remains an active area of investigation. Therefore, this study was designed to explore BLCA-4 expression and perform short tandem repeat (STR) genotyping using blood and urine samples from both smokers and non-smokers patients with bladder cancer, as well as from healthy controls. Additionally, cytological examination of urinary epithelial cells was conducted.

**Methods:**

A total of 273 participants were enrolled and stratified into three distinct groups: healthy controls with no smoking history (HPG, n = 70); patients diagnosed with bladder cancer with no smoking history (BC-S, n = 101); and patients diagnosed with bladder cancer with a positive smoking history (BC+S, n = 102).

**Results:**

This study demonstrates that the mean BLCA-4 concentration was significantly (P < 0.01) elevated in BC-S and BC+S groups relative to the HPG group across both blood and urine analysis. In the BC-S samples, a low-grade urothelial tumor was detected. In contrast, a high-grade urothelial tumor characterized by multiple large, isolated malignant cell structures was found in the BC+S samples. The results indicated changes at four loci (CSF1PO, D21S11, D7S820, and D2S1338); in contrast, the other five loci (Amelogenin, D13S317, D16S539, D18S51, and FGA) remained unchanged in LOH and MSI analysis across STR loci in urine samples from the BC-S and BC+S groups.

**Conclusions:**

Urine samples may be practically employed for diagnosing and predicting BC could be practically employed for STR genotyping to estimate the impact of smoking in patients with BC.

## 1 Introduction

Bladder cancer (BC) is defined as a malignant neoplasm originating from the urothelial cells lining the bladder (1). Histopathologically, Urothelial Carcinoma constitutes the predominant histological subtype, accounting for approximately 90% of cases. Other subtypes, such as Squamous Cell Carcinoma or Adenocarcinoma, may also occur, albeit less frequently (2). Globally, BC is ranked among the top ten most common diagnosed malignancies (3). According to GLOBOCAN international database, there were an estimated 549–393 new cases diagnosed in 2018, with 199–922 disease-specific mortality. Notably, the incidence rates of BC are demonstrated elevated within the Saudi Arabia population (4).

Smoking is established as the primary modifiable risk factor for urothelial carcinoma of the bladder. The carcinogens present in tobacco products induce malignant transformation in the urothelium, resulting in a 2-to-4-fold increase in relative risk among smokers compared to non-smokers (5). Epidemiological data attribute approximately 50% of BC cases in men and 30% in females to tobacco exposure, with a clear dose-response relationship correlating with both the intensity and duration of smoking (6, 7). The pathophysiological mechanism involves tobacco-derived carcinogens that induce DNA damage in the bladder epithelium, subsequently correlating with higher rates of disease recurrence (8). Furthermore, smoking modulates the tumor microenvironment (TME), thereby promoting immune evasion and potentially diminishing the clinical efficacy of immunotherapeutic interventions (9).

BLCA-4 is a nuclear protein specifically expressed in BC tissue, with its expression occurring early in the disease process (10). It acts as a transcription factor involved in regulating gene expression within malignant urothelial cells (11). A key characteristic of BLCA-4 is its presence in both tumor tissue and histologically normal urothelium obtained from patients with BC, underscoring its potential role in early carcinogenesis (12, 13). Quantitative assessment of BLCA-4 in urine samples via enzyme-linked immunosorbent assay (ELISA) has demonstrated high diagnostic accuracy (11), with reported sensitivity ranging from 89% to 96.4% and specificity between 95% and 100% (14). This renders BLCA-4 as a reliable, non-invasive biomarker for bladder cancer screening and surveillance (15). Furthermore, it has demonstrated superior diagnostic performance across all tumor grades compared with conventional urinary cytology and other urine-based biomarkers, such as BLCA-1 (16).

Short tandem repeats (STRs) are among the most important genetic markers in DNA research, and their variability among individuals makes them useful in genotyping and forensic investigations (17). Initially regarded as “non-functional” DNA, their regulatory roles in illnesses, including cancer, are now acknowledged (18). STR research reveals genomic instability in a variety of malignancies, including bladder cancer, where it can detect replication errors and genetic changes in urine samples (19).

Ultimately, BC is one of the malignancies most strongly associated with tobacco smoking (20, 21). However, much of the existing research in this domain has focused primarily on epidemiological data (22, 23), and has investigated BLCA-4, STR instability, or cytology individually in bladder cancer. The added value of our multimodal approach includes: (1) the integration of the protein biomarker (BLCA-4) with genomic instability (LOH/MSI) and morphological changes (cytology) within the same patient; (2) providing a more comprehensive molecular picture of smoking-related bladder cancer; and (3) demonstrating that urine as a non-invasive sample, can simultaneously serve all three analyses. Thus, we hypothesized that bladder cancer patients who smoke exhibit abnormal BLCA-4 levels in blood and urine, as well as an increased rate of LOH and MSI changes at specific STR sites, compared to non-smoking bladder cancer patients. Therefore, this study was designed to investigate: (a) BLCA-4 expression and STR genotyping in blood and urine samples; (b) cytological analysis of exfoliated urothelial cells; and (c) comparing the results among healthy non-smokers (HPG), non-smoking bladder cancer patients (BC-S), and smoking bladder cancer patients (BC+S).

## 2 Materials and methods

### 2.1 Ethical approval and participant consent

The study was conducted in accordance with the ethical standards established by the Deanship of Scientific Research of Princess Nourah bint Abdulrahman University. All experimental procedures and protocols were performed in compliance with relevant guidelines and regulations. The research project received official approval from the national ethics committee, the King Abdulaziz City for Science and Technology (KACST) in Riyadh, Saudi Arabia (study number H-01-R059, IRB log number 20-0242). Moreover, written informed consent was obtained from all participants prior to the collection of any human samples or data.

### 2.2 Experimental design

A total of 273 participants were enrolled and stratified into three distinct groups: healthy controls with no smoking history (HPG, n = 70), patients diagnosed with bladder cancer with no smoking history (BC-S, n = 101), and patients diagnosed with bladder cancer with a positive smoking history (BC+S, n = 102). Upon enrollment, self-reported demographic and clinical data were collected, which are presented in **Table 1**. Clinical parameters documented included the prevalence (%) of comorbidities such as nephropathy, nephrolithiasis (kidney stones), urinary tract disorders, peripheral arterial disease, hypertension, and diabetes mellitus. Additionally, family cancer history (%) was recorded. Baseline physiological measurements, including urinalysis parameters (mean ± SD), were assessed for all participants, assessing the following parameters: pH, uric acid (mg/day), ammonia (mmol/day), urea (g/day), and the presence of hematuria. These variables served as comparative metrics across the study groups.

**Table 1:** Demographic and medical information about study’s participants.

Covariate	HPG	BC-S	BC+S
No. of participants	70	101	102
Diagnostic age n (%)			
< 60 years	9/70 (12.85%)	56/101 (55.45%)	61/102 (59.80%)
≥ 60 years	61/70 (87.14%)	45/101 (44.55%)	41/102 (40.20%)
Comorbidities n (%)			
Nephropathy	0/70 (0%)	0/101 (0%)	4/102 (3.92%)
Nephrolithiasis	1/70 (1.43%)	4/101 (3.96%)	6/102 (5.88%)
Urinary tract disease	0/70 (0%)	8/101 (7.92%)	2/102 (3.70%)
Peripheral arterial disease	0/70 (0%)	5/101 (4.95%)	27/102 (26.47%)
Hypertension	0/70 (0%)	8/101 (7.92%)	63/102 (61.76%)
Diabetes mellitus	0/70 (0%)	13/101 (12.87%)	63/102 (61.76%)
Family cancer history n (%)			
Yes	0/70 (0%)	6/101 (5.94%)	0/102 (0%)
No	70/70 (100%)	95/101 (94.06%)	102/102 (100%)
Smoking history n (%)			
Never	70/70 (100%)	101/101 (100%)	0/102 (0%)
Former smokers (≥30 years)	0/70 (0%)	0/101 (0%)	102/102 (100%)
Urinalysis parameters (mean ± SD			
pH	5.82 ± 0.93	6.3 ± 1.17	7.7 ± 1.89
Uric acid (mg/day)	792.2 ± 3.75	801.9 ± 2.16	812.3 ± 1.78
Ammonia (mmol/day)	41.6 ± 2.47	54.2 ± 3.57	62.3 ± 2.53
Urea (g/day)	10.6 ± 1.56	14.3 ± 2.48	15.7 ± 1.84
Hematuria score	0	41.5 ± 2.24	55.7 ± 3.32

HPG = healthy controls with no smoking history (n = 70), BC-S = patients diagnosed with bladder cancer and no smoking history (n = 101), and BC+S = patients diagnosed with bladder cancer with positive smoking history (n = 102).

### 2.3 Blood and urine samples

Venous blood samples were collected from all participants in the morning, between 8:00 and 10:00 AM, to standardize conditions and minimize circadian variations. A total of 10 mL of peripheral blood was drawn from an appropriate antecubital vein following strict aseptic technique. The venipuncture site was first cleansed with a 70% alcohol swab and allowed to air-dry contamination and hemolysis. Blood samples were processed within two hours of collection, DNA was extracted using a spin column-based method following incubation with protease and buffer, followed by ethanol precipitation. In 200 µL of buffer, DNA was eluted. Since direct DNA extraction from whole blood is appropriate for STR genotyping, cutting down on processing time and degradation, peripheral blood mononuclear cells (PBMCs) were not isolated. The second tube was a gold-top serum separator tube (SST), which contained a clot activator and a gel barrier; this tube was used to obtain serum for BLCA-4 level estimation via enzyme-linked immunosorbent assay (ELISA). BLCA-4 levels were quantified using the Human BLCA-4 ELISA Test Kit (MyBioSource, San Diego, CA, USA, Catalog No. MBS2701541). This test employs a monoclonal antibody for capture and a biotin-labeled polyclonal antibody for detection, with a sensitivity of 0.078 ng/mL. The SST tubes were centrifuged at 3000 × g (gravitational force) for 15 minutes. The separated serum was then carefully aliquoted into sterile cryovials and stored at -20 °C until further biochemical analysis was performed. On the same morning, midstream clean-catch urine samples were collected from each participant using sterile, preservative-free containers. Prior to collection, all participants received both verbal and written instructions on the correct technique to minimize contamination. To ensure the accuracy of urinalysis, all tests were performed on the samples immediately after collection, within one hour. The BLCA-4 Urinary Tumor Marker Bioassay ELISA Kit was used for the quantitative identification of the BLCA-4 marker in human samples. Data were analyzed according to tumor categories, sizes, types, and clinical phases as outlined by the AJCC. Noninvasive stages (Ta and Tis) were defined as lacking metastasis, while invasive stages (T1-T4) indicated varying degrees of carcinoma affecting connective tissues and spreading to lymph nodes and other organs (24).

### 2.4 Polymerase chain reaction, and short tandem sequence

DNA was extracted using the QIAamp^®^ DNA Mini Kit (Cat. No. 51306, Qiagen, Hilden, Germany). Furthermore, DNA concentration and quality were assessed using a NanoDrop™ spectrophotometer (Thermo Fisher Scientific, USA). Genomic loci were subsequently amplified by PCR using the AmpFlSTR^®^ MiniFiler™ Amplification Kit. This kit allows simultaneous multiplex amplification of eight Short tandem sequence (STR) loci: D7S820, D13S317, D21S11, D2S1338, D18S51, D16S539, FGA, and CSF1PO. Additionally, the kit included the amelogenin locus for sex determination (25). All PCR reactions were performed according to the manufacturer’s protocol using a GeneAmp^®^ PCR System 9700 thermal cycler. The AmpFlSTR^®^ and MiniFiler™ Control DNA 007 were amplified as a positive control, and alleles were designated according to the provided allelic ladder. The genotype of the STR repeats was determined using capillary electrophoresis (CE) to isolate and identify STR alleles, using an ABI 3130 genetic analyzer. Each reaction involved a specific formulation, including 8.6 μL of Hi-Di formamide, 0.4 μL of GeneScan™ 500 LIZ size standard, and 1.0 μL of PCR product, 10 μL per sample in a 96-well genotyping plate Samples were heated at 95 °C for three minutes, rapidly cooled on ice for three minutes, and subsequently analyzed (**Table 2**). All samples were analyzed using the GeneMapper^®^ ID-X software (version 1.1). STR allele frequencies were calculated using GenAlEx V.6.503.

**Table 2:** PCR thermal cycling conditions.

Parameter	Initial denaturation	Denaturation	Annealing	Extension	Final extension	Final hold
Cycle #	Cycle (1)	Cycles (30)			Cycle (1)	
Temperature	95 °C	94 °C	95 °C	59 °C	72 °C	4 °C
Time	11 min	20 sec	2 min	1 min	45 min	∞ (hold)

### 2.5 Urinary cytology

All urine samples were examined. The samples were centrifuged at 250 × g for 10 minutes to pellet the sediment. The supernatant was discarded, and the sediment was resuspended by gentle vortexing. Two drops of the resuspended precipitate were placed on glass slides (26). The slides were immediately fixed in 96% ethanol for 30 minutes to prevent distortion caused by air drying. The slides were then stained using the standard Papanicolaou technique (27). All smears were examined independently by senior pathologists, who were blinded to clinical data. A total of 273 urine samples were examined and classified into categories: positive (low-grade and high-grade urothelial tumors) and negative (urothelial cell structures without abnormalities). With respect to tumor stage manifestation, these categories corresponded to noninvasive BC (Tis, Ta), invasive BC (T1, T2, T3), and metastatic BC (T4).

### 2.6 Statistical analysis

Statistical analyses were performed using Statistical Package for Social Sciences (SPSS for Windows, version 25; SPSS Inc., Chicago, IL, USA). The normality of data distribution was determined using the Shapiro-Wilk test. For normally distributed data (blood BLCA-4 levels), one-way ANOVA with Tukey’s *post-hoc* test was performed, followed by Duncan’s multiple range tests, according to the general linear model Y_ij_ = µ + T_i_ + A_j_ + E_ij,_ where Y_ij_ = experimental observation, µ = general mean, Ti = groups (i = CG, BC-S, and BC+S), and Eij = experimental error. For data that were not normally distributed (urine BLCA-4 levels), the Kruskal-Wallis’s test was used. The Chi-squared test was performed to determine STR mutation frequencies. A P-value below 0.05 was considered statistically significant.

## 3 Results

### 3.1 Serum BLCA-4 marker

**Figure 1:**
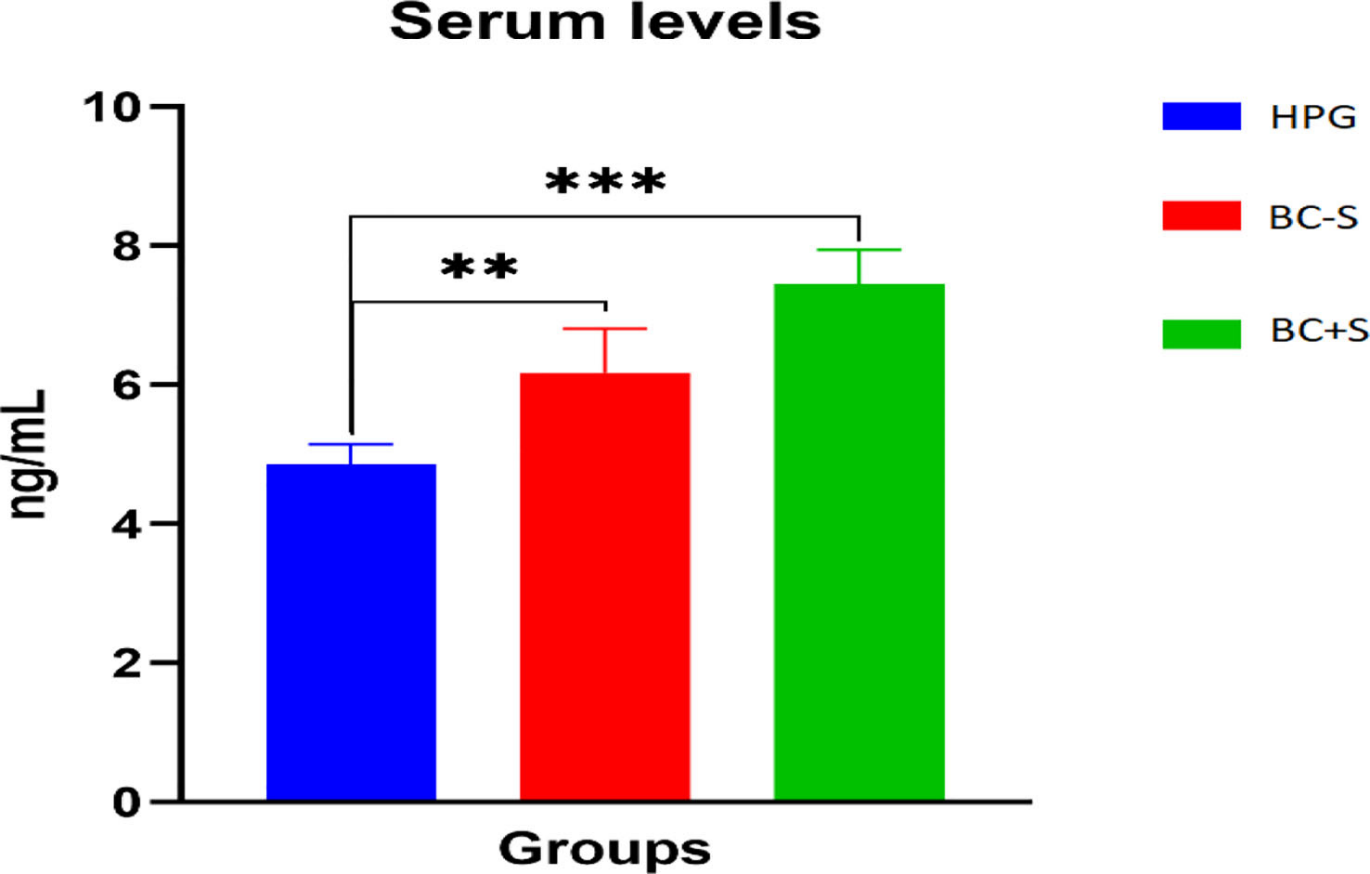
Scopes of BLCA-4 in healthy controls with no smoking history (HPG; n = 70), patients diagnosed with bladder cancer and no smoking history (BC-S; n = 101), and patients diagnosed with bladder cancer with positive smoking history (BC+S; n = 102). Numbers are placed as (mean ± SEM, n = 50) of three trials. ***P < 0.001, **P < 0.01 (ng/mL).

Blood analysis demonstrated that the mean BLCA-4 concentration was significantly elevated in both bladder cancer groups (BC-S and BC+S) compared to the HPG (**Figure 1**). The BC+S group showed a significant increase (P < 0.01), with a mean concentration of 7.452 ± 0.49 versus 4.856 ± 0.29 ng/mL in the HPG. Furthermore, the BC-S group showed a highly significant elevation in median BLCA-4 levels (P < 0.01), with a mean concentration of 6.180 ng/mL ± 0.63 compared to 4.856 ng/mL ± 0.29 in the HPG (**Figure 1**).

**Figure 2:**
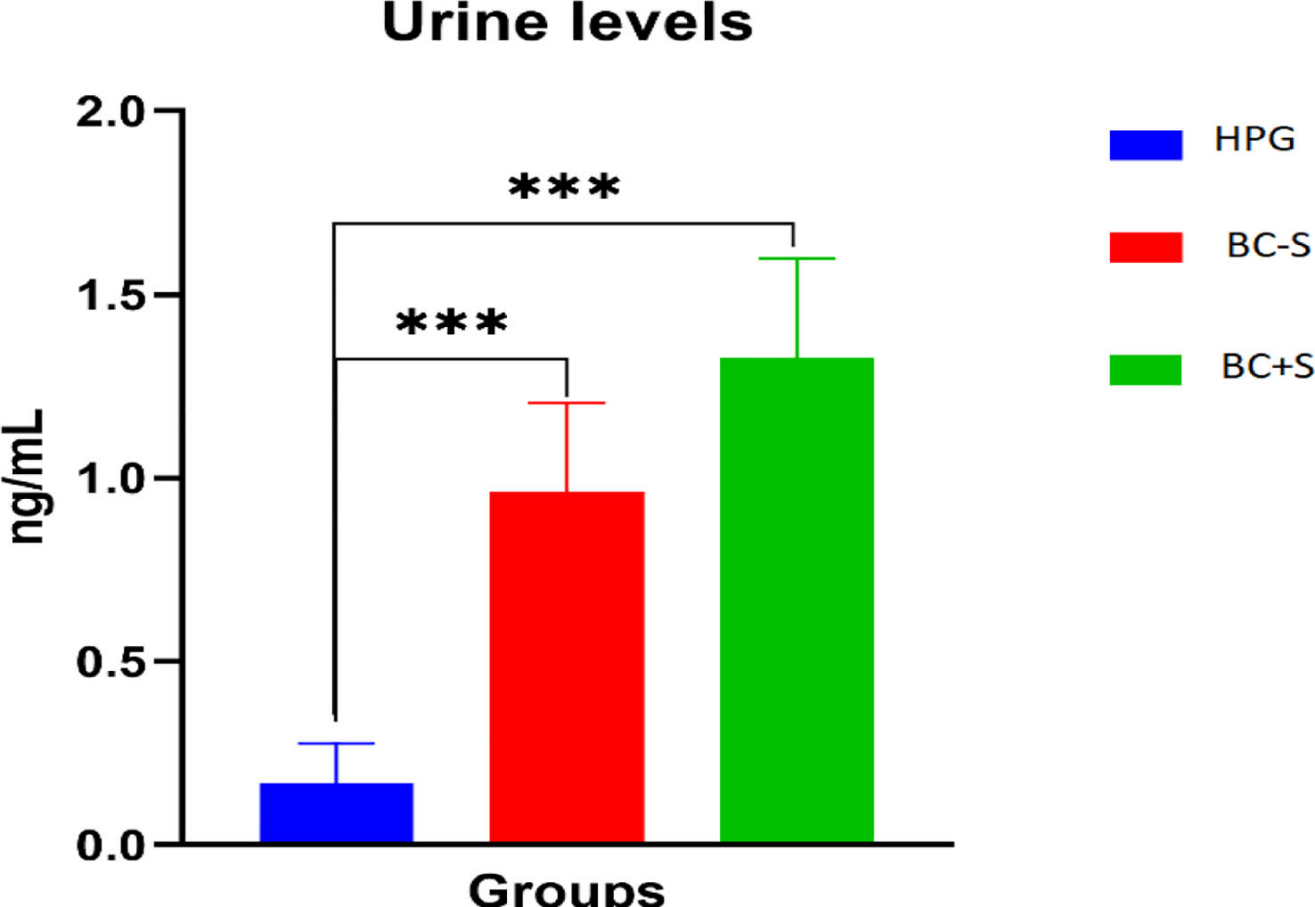
Scopes of BLCA-4 in urine in healthy controls with no smoking history (HPG; n = 70), patients diagnosed with bladder cancer and no smoking history (BC-S; n = 101), and patients diagnosed with bladder cancer with positive smoking history (BC+S; n = 102). Numbers are placed as (mean ± SEM, n = 50) of three trials. ***P < 0.001, (ng/mL).

In contrast, urine analysis showed that the mean BLCA-4 concentration was significantly elevated in both BC-S and BC+S groups compared to the HPG (**Figure 2**). The BC+S group exhibited significantly higher median BLCA-4 levels (1.328 ± 0.27 ng/mL) than HPG (0.168 ± 0.11 ng/mL; P < 0.01). Similarly, the BC-S group showed significantly higher median BLCA-4 levels (P < 0.01), with 0.964 ng/mL ± 0.24 compared to 0.168 ± 0.11 ng/mL in the HPG (**Figure 2**).

### 3.2 Tumor typification and cytological analysis of urothelial cell structures

**Figure 3:**
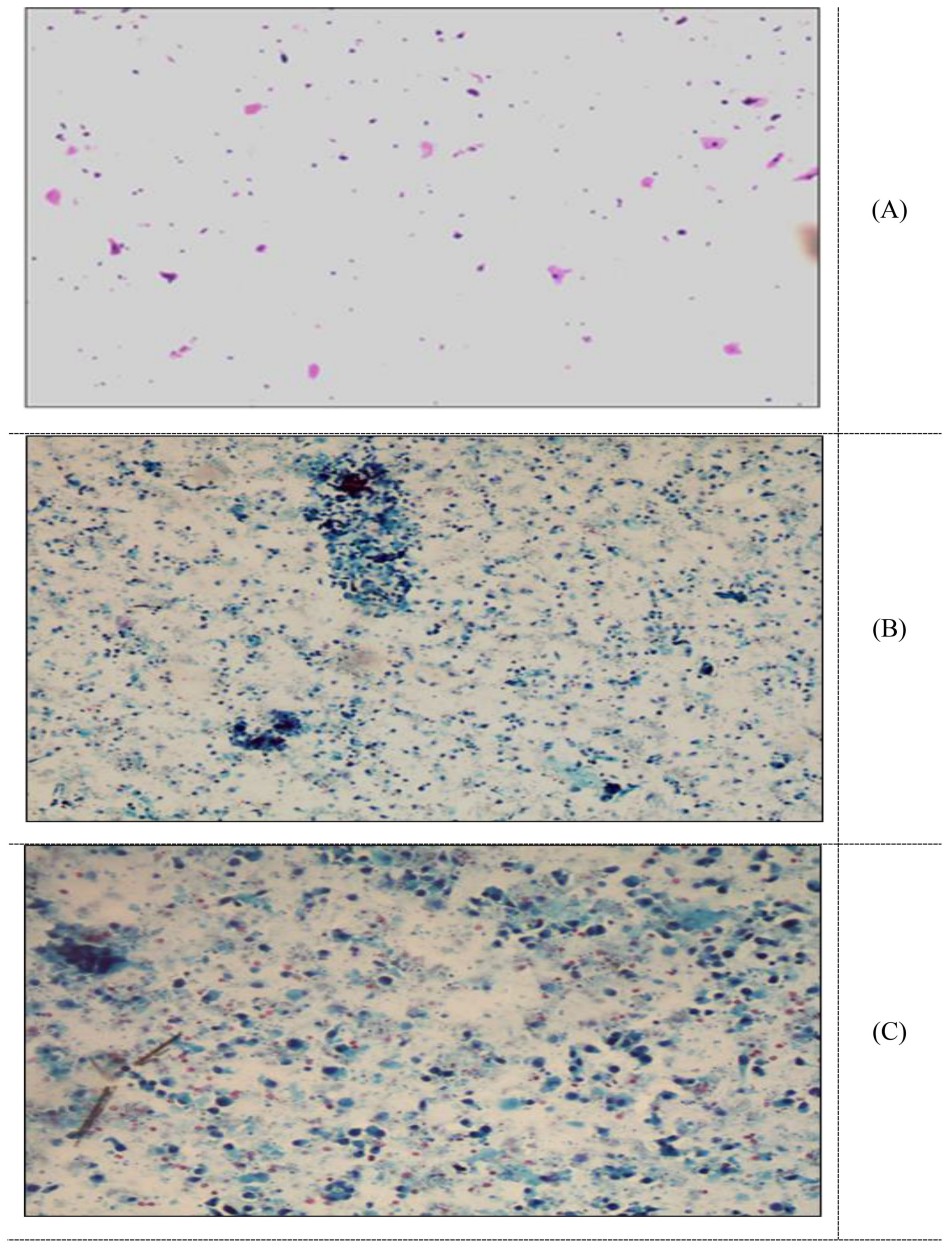
Urothelial cells with Papanicolaou stain, zooming x 20 in **(A)** healthy controls with no smoking history (HPG; n = 70), **(B)** patients diagnosed with bladder cancer and no smoking history (BC-S; n = 101), and **(C)** patients diagnosed with bladder cancer with positive smoking history (BC+S; n = 102).

**Figure 4:**
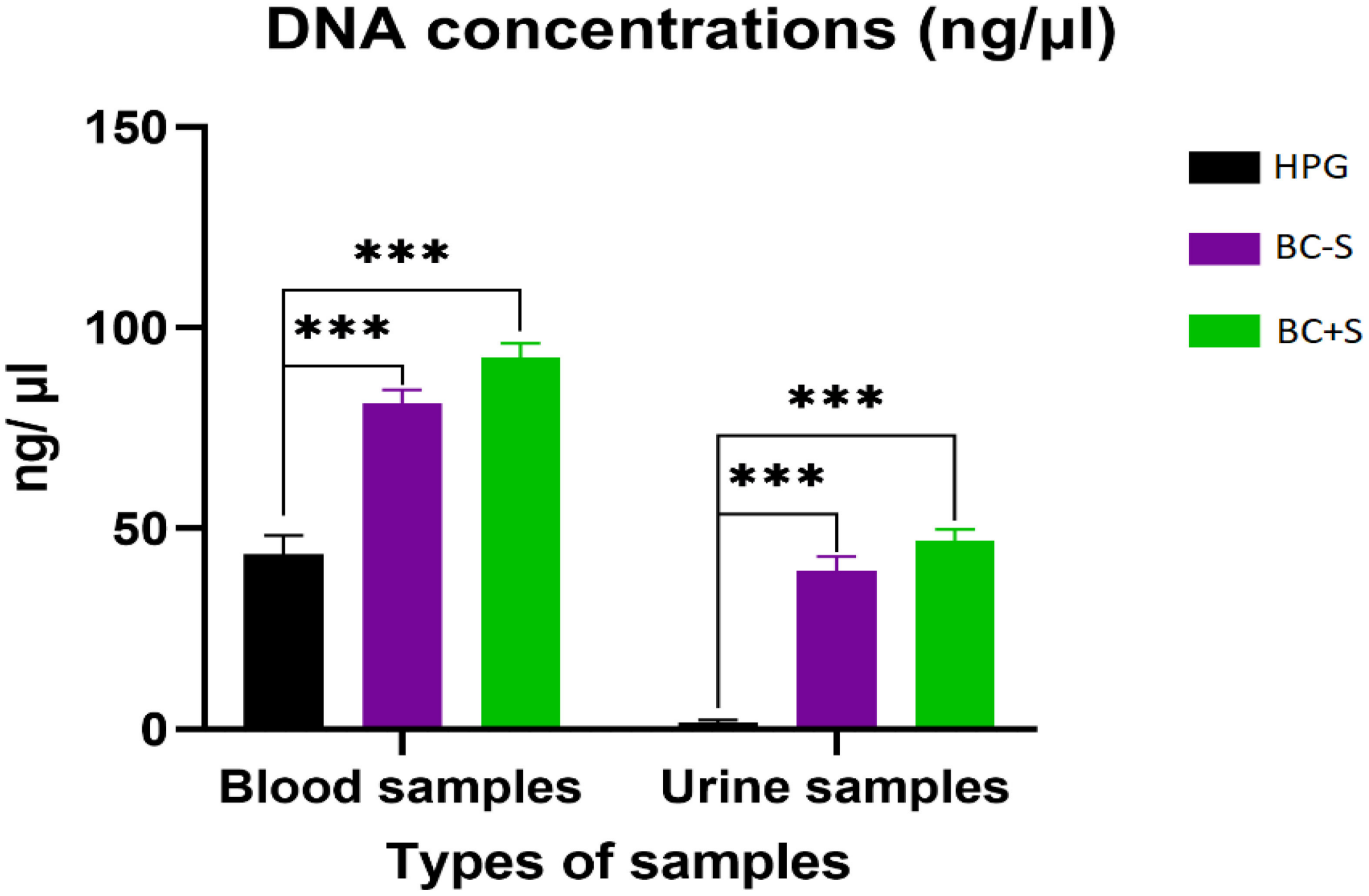
DNA contents in urine and blood in healthy controls with no smoking history (HPG; n = 70), patients diagnosed with bladder cancer and no smoking history (BC-S; n = 101), and patients diagnosed with bladder cancer with positive smoking history (BC+S; n = 102). Numbers are placed as (mean ± SEM, n = 50) of three trials. ***P < 0.001.

Microscopic analysis of urothelial cells in the HPG showed single, flat, small, and scattered cell structures. Several cells were umbrella type, relating to the bladder, although all were detected in limited numbers (**Figure 3**). Furthermore, a low-grade urothelial tumor was present in the BC-S sample, and its characteristics were associated with cytoplasmic homogeneity, an increased nuclear-to-cytoplasmic ratio, a predominance of small cell clusters, occasional multinucleation, and numerous red cells (hematuria) with occasional cell clusters (**Figure 4**).

**Figure 5:**
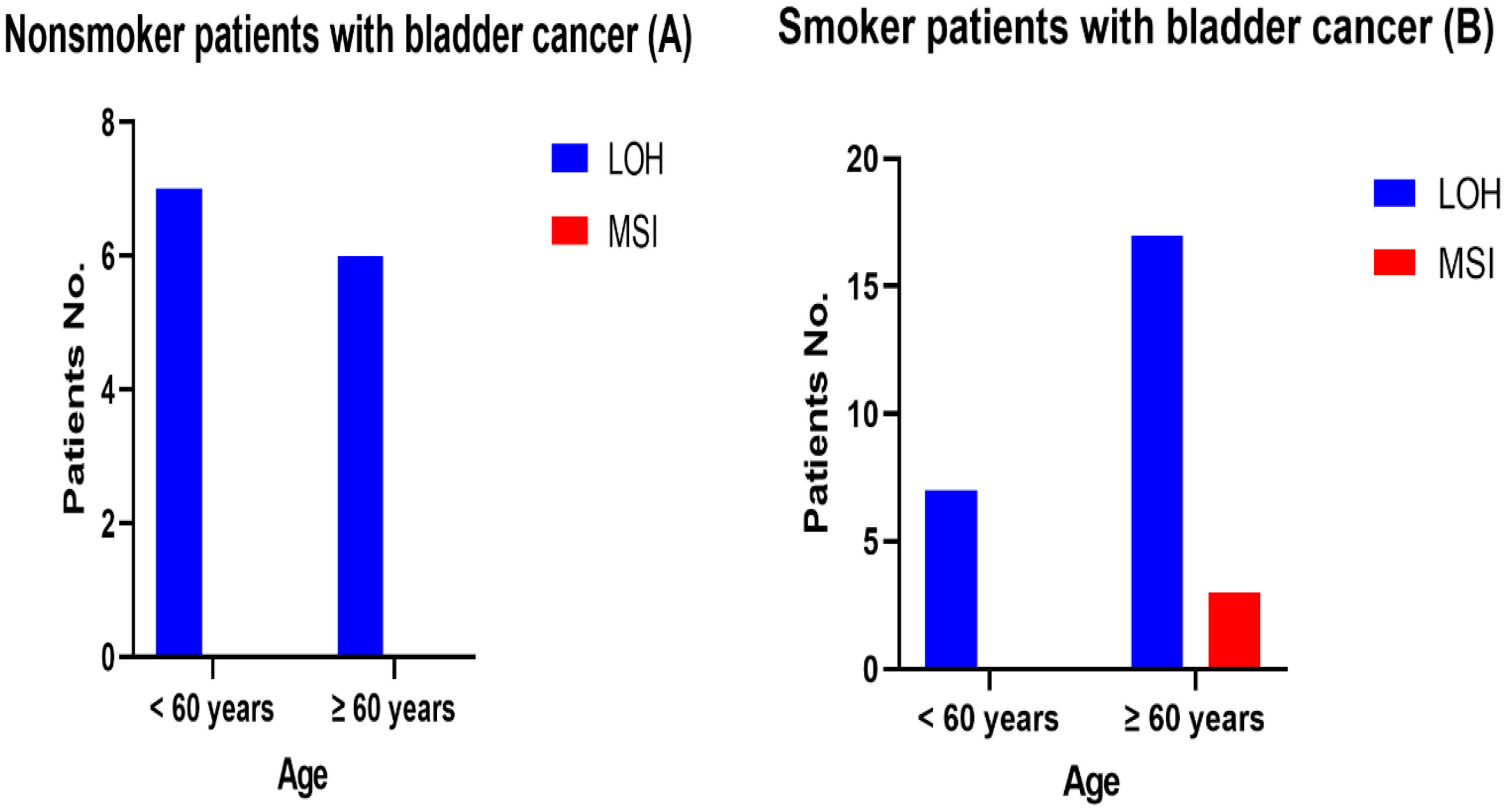
Relationship between mutations LOH and MSI within STR loci and variables of age in BC-S: Bladder cancer patients with no history of smoking; BC+S: Bladder cancer patients with a positive history of smoking.

In contrast, a high-grade urothelial tumor was present in the BC+S sample, and its characteristics were associated with increased nuclear-to-cytoplasmic ratio, multiple large, isolated malignant cell with evident nuclear hyperchromasia, coarse chromatin, multinucleation, and variably sized nucleoli within papillary architectural fragments (**Figure 5**).

As shown in **Table 3**, tumors in the BC+S group were more frequently multifocal, with multiple tumors identified in 54 out of 102 patients (52.9%), compared with only 16 of 101 patients (15.8%) in the BC-S group.

**Table 3:** Clinicopathological characteristics of bladder tumors stratified by smoking status in patients with bladder cancer (BC).

Characteristic	BC-S^*^	BC+S^*^
Tumor multiplicity		
Single	85/101 (84.15%)	48/102 (47.06%)
Multiple	16/101 (15.84%)	54/102 (52.94%)
Tumors size		
< 3 cm	87/101 (86.13%)	65/102 (63.73%)
≥ 3 cm	14/101 (13.86%)	37/102 (36.27%)
Tumor grade		
Low grade	88/101 (87.12)	23/102 (22.55%)
High grade	13/101 (12.87)	79/102 (77.45%)
Pathological stage		
Noninvasive (Tis, Ta)	86/101 (85.14)	23/102 (22.55%)
Invasive (T1 - T3)	7/101 (6.93)	47/102 (46.08%)
Metastatic (T4)	8/101 (7.92)	32/102 (31.37%)

^*^Data are presented as n (%). BC-S: Bladder cancer patients with no history of smoking; BC+S: Bladder cancer patients with a positive history of smoking.

Regarding tumor size, lesions ≥ 3 cm were more prevalent in smokers, accounting for 37 cases (36.3%), whereas the majority of tumors in non-smokers were less than 3 cm (87 patients, 86.1%). Significant differences were also observed in tumor grade; high-grade tumors predominated in the BC+S group (77.5%), whereas low-grade tumors predominated in the BC-S group (87.1%). Moreover, the pathological stage at presentation varied considerably among the cohorts. Non-invasive diseases (Ta and Tis) represented the most common presentation in non-smokers (86/101, 85.1%). In contrast, smokers were more frequently presented with muscle-invasive or more advanced diseases, with invasive (T1-T3) and metastatic (T4) stages observed in 47 (46.1%) and 32 (31.4%) patients, respectively (**Table 3**).

### 3.3 Genetic evaluation

Blood and urine DNA concentrations for the HPG, BC-S, and BC+S groups are shown in **Table 4**. However, the BC-S group exhibited a significant difference between blood DNA and urine concentrations, with 81.27 ± 3.31 and 39.56 ± 3.44 ng/µL, respectively. In addition, the DNA concentrations in the BC+S group were 92.57 ± 3.64 ng/µL in blood and 46.97 ± 2.86 ng/µL in urine (**Table 4**). Overall, DNA concentrations in blood and urine samples were higher in BC-S and BC+S groups compared to the HPG (**Figure 4**).

**Table 4:** Mean DNA concentrations in all blood and urine samples from all participants across the three cohorts.

Study groups	Blood (ng/µL)	Urine (ng/µL)	P-value
HPG	43.73 ± 4.62	1.74 ± 0.61	<0.000427
BC-S^*^	81.27 ± 3.31	39.56 ± 3.44	<0.000159
BC+S ^*^	92.57 ± 3.64	46.97 ± 2.86	<0.000925

*Data are presented as (ng/µL). BC-S: Bladder cancer patients with no history of smoking; BC+S: Bladder cancer patients with a positive history of smoking. Independent t-test (2-tailed) for ongoing factors, Pearson’s X2 test for categorical factors.

Molecular analysis of 2,457 STR loci across nine autosomal markers in 101 urine samples from the BC-S group and 102 urine samples from the BC+S group is summarized in **Table 5**. The BC+S group showed a substantially higher (P < 0.01) incidence of loss of heterozygosity (LOH; 0.8547%) than the BC-S group (0.5291%). Moreover, microsatellite instability (MSI) was detected in the BC+S group at a rate of 0.2442% but was absent in BC-S samples. Smokers showed a considerably higher total mutation rate (1.0989%) than non-smokers (0.5291%), indicating an association between smoking history and genomic instability (P < 0.01). The results indicated changes at four loci: CSF1PO, D21S11, D7S820, and D2S1338; in contrast, the remaining five loci (Amelogenin, D13S317, D16S539, D18S51, and FGA) showed no changes in LOH and MSI analysis across STR loci in urine samples from the BC-S and BC+S groups (**Table 6**). Moreover, the D21S11 locus was the most susceptible among those examined, whereas the D2S1338 locus was the least susceptible to LOH-related deviations. The D7S820 locus showed a mutation rate of 0.122% in the BC-S group (3 LOH events) compared with 0.284% in the BC+S group (6 LOH and 1 MSI event; P < 0.01). The CSF1PO locus exhibited a mutation rate of 0.081% in the BC−S group (2 LOH events) compared with 0.162% in the BC+S group (4 LOH events; P < 0.01).

**Table 5:** LOH and MSI mutation frequencies found in total 2457 loci for nine autosomal STR loci and in 203 urine specimens derived from BC-S, and BC+S.

Mutation type	No. of mutations in BC-S	Mutation frequency (%)	Number of mutations in BC+S	Mutation frequency (%)	P value
LOH	13	0.5291	21	0.8547	<0.000851
MSI	0	0	6	0.2442	<0.000141
Total	13	0.5291	27	1.0989	<0.000421

*BC-S: Bladder cancer patients with no history of smoking; BC+S: Bladder cancer patients with a positive history of smoking. ***P < 0.001. loss of heterozygosity (LOH), microsatellite instability (MSI).

**Table 6:** Distribution of LOH/MSI alterations identified in all 2457 loci for nine STR loci and in 203 urine specimens derived from BC-S, and BC+S.

STR Locus	Chromosome #	Mutation rate (%)	No. of deviations in BC-S		Total mutation/deviation rate (%)	No. of deviations in BC+S		P-value
			LOH	MSI		LOH	MSI	
Amelogenin	Sex	0.00	0	0	0.00	0	0	ns
D13S317	13	0.00	0	0	0.00	0	0	ns
D16S539	16	0.00	0	0	0.00	0	0	ns
D18S51	18	0.00	0	0	0.00	0	0	ns
CSF1PO	5	0.081	2	0	0.162	4	0	<0.000141
D21S11	21	0.284	7	0	0.569	12	2	<0.000741
D7S820	7	0.122	3	0	0.284	6	1	<0.000524
FGA	4	0.00	0	0	0.00	0	0	ns
D2S1338	2	0.040	1	0	0.081	2	0	<0.000145
Total		0.5291	13		1.0989	27		<0.000214

*BC-S: Bladder cancer patients with no history of smoking; BC+S: Bladder cancer patients with a positive history of smoking. ***P < 0.001. loss of heterozygosity (LOH), microsatellite instability (MSI), ns = not significant.

However, the D2S1338 locus showed the lowest mutation frequency, with 0.040% in the BC−S group and 0.081% in the BC+S group. MSI was detected only in the BC+S group, specifically at the D21S11 and D7S820 loci. In total, 13 LOH mutations were identified in the BC−S group, whereas 27 mutations (24 LOH and 3 MSI) were detected in the BC+S group, indicating a significantly higher total mutation rate in smokers (1.0989%) than in non-smokers (0.5291%). **Figure 5** demonstrates the relationship between deviation type within STRs and age variables in nonsmokers with BC (**Figure 5A**) and smokers with BC (**Figure 5B**).

## 4 Discussion

Given the well-established association between tobacco smoking and bladder cancer (20–23), this study investigated the relationship between tobacco-related carcinogens and bladder cancer through identifying BLCA-4 markers and analyzing short tandem repeats (STRs) as markers of microsatellite repeat inactivation. Blood and urine samples were obtained from smokers and non-smokers diagnosed with bladder cancer and from a control group of healthy individuals. Additionally, cytological examination of urothelial cells was conducted.

Marked differences were observed in urine parameters among participants in BC-S and BC+S compared to the HPG including pH, uric acid, ammonia, urea, and hematuria in urine. According to Van et al. (28) reported that smoking tobacco was associated with differences resulted in distinctions in urine tests among ex-prisoners.

The presented study showed that the mean BLCA-4 concentration was significantly elevated in BC-S and BC+S compared to the HPG in blood and urine analysis. Most BC patients (75.3%) were either current or former smokers, and their blood and urine samples had considerably higher levels of BLCA-4 than those of the control group, according to Szymańska et al. (29). In addition, assessments of BLCA-4 have revealed high diagnostic accuracy, with a sensitivity ranged from 89% to 96.4% and a specificity ranged from 95% to 100% (14). This renders BLCA-4 a valuable non-invasive biomarker for bladder cancer screening and surveillance (15, 30).

Serum is a superior source of DNA compared with urine, as it contains a higher cellularity (31–33). Overall, DNA content in blood and urine samples was higher in BC-S and BC+S groups than in HPG. Cytological samples from the BC-S showed a low-grade urothelial tumor, whereas those from the BC+S samples showed high-grade urothelial tumor with multiple large, isolated malignant cells. The present findings are consistent with those of other studies on the impact of smoking on urothelial transitional cell tumors, concluding that smoking history is associated with high-level carcinomas, whereas non-smoking cases are associated with low-grade urothelial tumors (34–37). Moreover, Dimashkieh et al. (38) analyzed 1835 urine samples and reported increased urothelial cells in participants with BC, suggesting genetic alterations and pathological cell behavior.

The Implementation of the Paris System for Reporting Urinary Cytology (TPS) significantly improves diagnostic reproducibility and accuracy (39), TPS has been reported to have a sensitivity of 84.8% and a specificity of 91.3% for high-grade urothelial carcinoma, with improved inter-examiner agreement (40). On the other hand, a narrative review has highlighted that deep learning algorithms effectively assist in grading and identifying malignant features, achieving accuracy comparable to expert pathologists. Notably, deep learning models demonstrate increased sensitivity for high-grade urothelial carcinoma while maintaining specificity (41). AI algorithms demonstrate high performance in the automated identification of atypical cells, achieving > 90% accuracy in classifying cytology images classification (42). Future studies should incorporate digital pathology to enhance diagnostic accuracy and reduce inter-observer variability (43).

Short tandem repeats (STRs) are among the most important genetic markers in DNA research, and their variability among individuals makes them useful in genotyping and forensic investigations (17). In this study, blood and urine samples were evaluated using BLCA-4 as a urinary tumor marker and subjected to DNA assessments, including STR genotyping at nine loci. The results showed changes at four loci: CSF1PO, D21S11, D7S820, and D2S1338. In contrast, blood tests revealed complete STR genotypes in all participants, consistent with the findings of Itoh et al. (44). In contrast, five loci (Amelogenin, D13S317, D16S539, D18S51, and FGA) showed no changes in LOH and MSI analysis in urine samples from the BC-S and BC+S groups. However, STR analysis reveals genomic instability in a variety of malignancies, including BC, and can detect replication errors and genetic changes in urine samples (19). Finally, initially regarded as “non-functional” DNA, STRs are now recognized to have regulatory roles in diseases, including cancer (18).

## 5 Limitations and future studies

1. Although ELISA is a reliable method for protein quantification, independent validation of BLCA-4 measurements remains essential, especially in tumor studies where assay specificity and reproducibility are of paramount importance.
2. One limitation of this study was the absence of independent validation for BLCA-4 measurements, given that the study solely relied on ELISA. Therefore, we recommend the use of Western blotting, a recognized quantitative validation method, for independently corroborating the findings and excluding any possible cross-reactivity.
3. This study also utilized a limited set of nine STR loci, which are less accurate than next-generation sequencing (NGS)-based DNA mismatch repair assays. Although this method was practical and cost-effective in this pilot study, it likely detects only a small fraction of the genetic alterations associated with smoking.
4. Future research should explore quantitative real-time polymerase chain reaction (qRT-PCR) for measuring BLCA-4 gene expression in urine sediments, whereas the current study focused on protein level measurement by. This approach may improve the sensitivity of non-invasive BC screening and yield additional genetic information (45).
5. The integration of these markers into diagnostic algorithms may enhance sensitivity without compromising specificity, particularly in the monitoring patients with low-grade, non-invasive BC, for whom cytology is less effective (46, 47).

## 6 Conclusion

This study investigated BLCA-4 expression and performed short tandem repeat (STR) genotyping using blood and urine samples from bladder cancer patients, including both smokers and non-smokers, in addition to cytological evaluation of exfoliated urothelial cells. These findings are consistent with current evidence regarding diagnostic and prognostic strategies in BC and highlight the role of BLCA-4 expression and STR genotyping in assessing smoking-related cellular damage and patient outcomes. However, future research should prioritize orthogonal validation of ELISA results by using complementary protein detection methods such as Western blotting, rather than relying on a single analytical platform. Additionally, subsequent studies should employ next-generation sequencing (NGS)-based assays in place of the limited nine-locus STR panel. Comprehensive genomic profiling and quantitative real time PCR (qRT-PCR) analysis for BLCA-4 transcript levels are also warranted. The integration of these multimodal biomarkers into diagnostic algorithms may enhance sensitivity, particularly for the surveillance of low-grade, non-muscle-invasive BC.

## Data Availability

The original contributions presented in the study are included in the article/supplementary material. Further inquiries can be directed to the corresponding author.
